# Oncometabolites: tailoring our genes

**DOI:** 10.1111/febs.13295

**Published:** 2015-04-30

**Authors:** Stefan Nowicki, Eyal Gottlieb

**Affiliations:** ^1^ Cancer Research UKBeatson InstituteGlasgowUK

**Keywords:** cancer, dioxygenase, epigenetics, metabolism, oncometabolites

## Abstract

Increased glucose metabolism in cancer cells is a phenomenon that has been known for over 90 years, allowing maximal cell growth through faster ATP production and redistribution of carbons towards nucleotide, protein and fatty acid synthesis. Recently, metabolites that can promote tumorigeneis by altering the epigenome have been identified. These ‘oncometabolites’ include the tricarboxylic acid cycle metabolites succinate and fumarate, whose levels are elevated in rare tumours with succinate dehydrogenase and fumarate hydratase mutations, respectively. 2‐Hydroxyglutarate is another oncometabolite; it is produced *de novo* as a result of the mutation of isocitrate dehydrogenase, and is commonly found in gliomas and acute myeloid leukaemia. Interestingly, the structural similarity of these oncometabolites to their precursor metabolite, α‐ketoglutarate, explains the tumorigenic potential of these metabolites, by competitive inhibition of a superfamily of enzymes called the α‐ketoglutarate‐dependent dioxygenases. These enzymes utilize α‐ketoglutarate as a cosubstrate, and are involved in fatty acid metabolism, oxygen sensing, collagen biosynthesis, and modulation of the epigenome. They include enzymes that are involved in regulating gene expression via DNA and histone tail demethylation. In this review, we will focus on the link between metabolism and epigenetics, and how we may target oncometabolite‐induced tumorigenesis in the future.

**Abbreviations**
2HG: 2‐hydroxyglutarate
5‐hmC: 5‐hydroxymethylcytosine
ACLY: ATP‐citrate lyase
ACSS2: acetyl‐coenzyme A synthetase short‐chain family member 2
AML: acute myeloid leukaemia
BCAT1: branched‐chain amino acid transferase 1
FH: fumarate hydratase
H3K27: histone H3 Lys27
H3K27me3: trimethylated histone H3 Lys27
H3K9: histone H3 Lys9
H3K9me3: trimethylated histone H3 Lys9
HAT: histone acetyltransferase
HDM: histone demethylase
HIF: hypoxia‐inducible factor
HMT: histone methyltransferase
IDH: isocitrate dehydrogenase
mTHF: methyltetrahydrofolate
PHD: prolyl hydroxylase
SAM: *S*‐adenylmethionine
SDH: succinate dehydrogenase
TCA: tricarboxylic acid
TET: 10‐11‐translocation methylcytosine dioxygenase
αKG: α‐ketoglutarate

## Cancer metabolism

It has now been over 90 years since it was first observed that cancer cells have an altered metabolic phenotype 1. In the presence of oxygen, normally differentiated cells predominantly utilize the tricarboxylic acid (TCA) cycle and oxidative phosphorylation to efficiently produce energy and the metabolites necessary for protein and lipid synthesis. However, in the presence of hypoxia, this process is altered, and cells switch to a higher rate of glycolysis and lactate production to maintain their energy and metabolic needs. In cancer cells, glycolysis is maintained at a high rate, even in the presence of oxygen; this is termed ‘aerobic glycolysis’. This seems to be counterintuitive, as oxidative phosphorylation is much more efficient at energy production than glycolysis, producing 34 more ATP molecules from the same molecule of glucose 2. Tumour cells, however, are rapidly dividing, and have a much greater need for anabolism than normally differentiated cells. Rapid glucose metabolism enables faster ATP production and greater redistribution of carbons to nucleotide, protein and fatty acid synthesis, thus maximizing cell growth 3. [^18^F]Deoxyglucose positron emission tomography utilizes this feature of cancer by allowing visualization of glucose uptake in patients, and has become an important tool in cancer diagnosis and the measurement of treatment response 4. It has been used to confirm the correlation between glucose metabolism and cell proliferation rate in some human tumours 5, 6, 7. Other malignancies, such as pancreatic cancer, do not show this relationship, probably because of complex interactions *in vivo* between the tumour and the microenvironment 8. Rapid glucose metabolism can also occur in normal cells, where there is a need for rapid growth and proliferation, such during an immune response, during wound healing, and *in utero* 9, 10, 11. In cancer cells, however, ‘aerobic glycolysis’ can be deregulated, in part because of genetic mutations, such as in the phosphoinositide 3‐kinase–AKT and Myc pathways 3. In addition, increased activation in cancer of a specific isoform of pyruvate kinase, PKM2, has been identified; this, because of its controlled enzymatic activity, can shift metabolic flux away from the TCA cycle to other anabolic processes 12. Recently, other metabolic changes, driven by mutations in genes related to the TCA cycle, have indicated an alternative role, that of the ‘oncometabolite’ 13. In this instance, a particular metabolite builds up within the cell and contributes to the tumorigenic process. Mutations in fumarate hydratase (FH) and succinate dehydrogenase (SDH) subunits follow the classic Knudson ‘two‐hit’ model, with loss of gene function, and accumulation of the substrates fumarate and succinate, respectively. Conversely, for isocitrate dehydrogenase (IDH), a single‐allele mutation confers a gain of function, producing an excess of a new metabolite, 2‐hydroxyglutarate (2HG) 13. These oncometabolites seem to have a common tumorigenic mechanism, namely the competitive inhibition of a superfamily of enzymes, the α‐ketoglutarate (αKG)‐dependent dioxygenases, which are important modulators of both the oxygen‐sensing machinery and the epigenome, providing a link between metabolic dysfunction and altered gene expression in cancer.

## Regulation of the epigenome

Over the past 20 years, almost in parallel with the increasing understanding of cancer metabolomics, great strides in our knowledge of the epigenome have been made. This constitutes a range of changes that occur to DNA expression without altering the DNA sequence. The chromatin structure is organized in several layers that can be modulated to alter gene expression. One hundred and forty‐seven base pairs of DNA are folded around a histone core, which comprises eight subunits, i.e. two each of histones H2A, H2B, H3, and H4, to form a nucleosome. Gene expression can be modulated epigenetically both at the DNA nucleotide level through cytosine methylation, and via chemical modification of histone tails within nucleosomes. These processes are essential in normal healthy tissues to maintain cell lineage and differentiation by activating and suppressing genes that are vital for cellular functions.

Changes in DNA methylation have long been known to occur in cancer 14. This process is modulated by DNA methyltransferases (DNMTs) and 10‐11‐translocation methylcytosine dioxygenase (TET), which, respectively, add and remove methyl groups. In fact, TET enzymes oxidize (hydroxylate) the methyl groups, resulting in their removal. Large areas of the genome of cancer cells are hypomethylated as compared with normal cells, specifically in gene‐poor regions, and are associated with chromatin changes leading to genomic instability 15. Conversely, short regions are hypermethylated, specifically at CpG islands. These sequences are over 200 bp long, with over 50% GC content, are usually found near promoter sites, and are associated with gene silencing 16. A subset of tumours in glioma and colorectal cancer have been identified with a specific CpG island methylator phenotype, whereby a large number of such loci are hypermethylated in a distinctive pattern 17, 18.

Gene expression can also be altered by changes in chromatin structure via chemical modification of amino acids on histone tails. This can determine the extent to which DNA is exposed to transcription factors, and this depends on the type of chemical change, and the degree and site of modification. A complex array of modifications can occur, commonly acetylation and methylation, but also ubiquitination, phosphorylation, ADP‐ribosylation, and SUMOylation 19. Acetylation is catalysed by histone acetyltransferases (HATs), on lysines, and is associated with increased gene expression. Conversely, histone deacetylases hydroxylate the acetyl group, causing its subsequent removal. Histone methylation, however, is more complex. Amino acids on histone tails, predominantly lysine, can receive up to three methyl groups by the action of a variety of histone methyltransferases (HMTs), with each of these modifications potentially conferring a different function 20. Histone modification is a dynamic process, so equally important is the activity of histone demethylases(HDMs), which, similarly to TET, catalyse the oxidation of methyl groups, resulting in their removal. Unlike acetylation, methylation can be associated with both activation and suppression of genes. Methylation of histone H3 Lys4, for instance, is associated with gene activation, whereas methylation of histone H3 Lys9 (H3K9) and methylation of histone H3 Lys27 (H3K27) are associated with gene silencing 21. These epigenetic changes have been shown to be important in human cancers, with global changes in histone acetylation and methylation levels having important prognostic significance in prostate, colon and non‐small‐cell lung cancer 22, 23, 24.

Drugs have been developed in an attempt to target these epigenetic hallmarks of cancer. DNMTs were the first to be targeted successfully. Decitabine and azacitidine were developed > 50 years ago, but were limited to the treatment of myelodysplastic syndromes, owing to toxic side effects at high doses 25. The only other drugs used in clinical practice are vorinostat and romidepsin, which inhibit histone deacetylases. Both drugs are used in the treatment of T‐cell lymphoma, but, disappointingly, they have also failed to show clinical efficacy in other tumour types, owing to toxicity 26. To date, other potential targets, such as HMTs, have not provided any meaningful clinical results. This difficulty in drug development may be attributable to the broad activity of these enzymes, which often also act on non‐nuclear proteins 27. Targeting the metabolic causes of these epigenetic changes may therefore have greater clinical potential.

## The metabolic mechanism of epigenetic change

**Figure 1:**
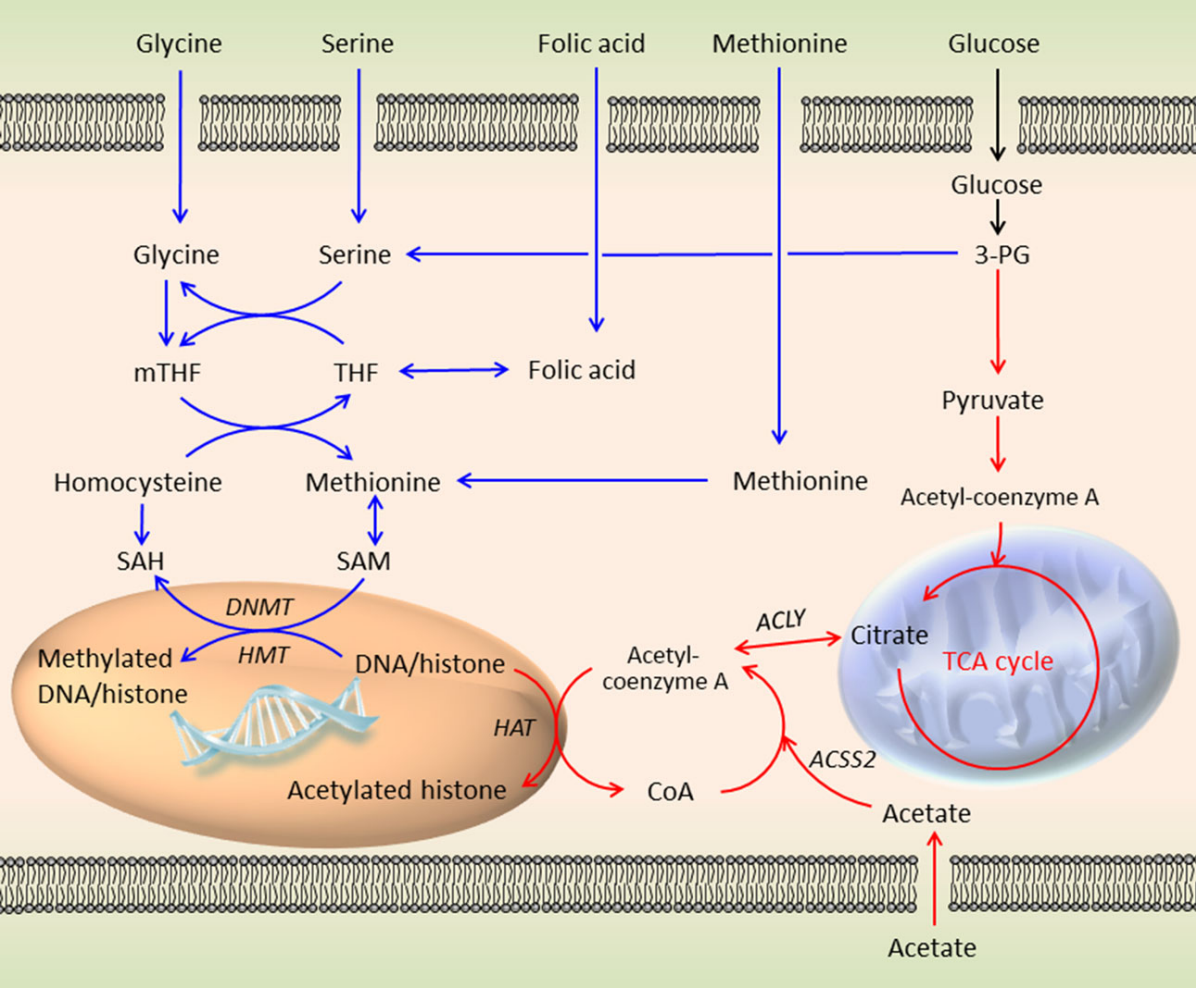
Methyl and acetyl transfer pathways. The blue pathway depicts one‐carbon metabolism and its generation of SAM, which provides a methyl group for histone and DNA methylation. Imported folate is reduced to tetrahydrofolate (THF) and subsequently methylated to mTHF by the conversion of serine to glycine and the glycine cleavage system. The folate cycle is coupled to the methionine cycle by mTHF, donating a carbon to homocysteine, converting it to methionine. Adenylation of methionine produces SAM, which acts as a cosubstrate for DNMT and HMT, allowing transfer of its methyl group to DNA and histone tails respectively. The red pathway depicts acetyl transfer from acetyl‐coenzyme A. Acetyl‐coenzyme A, which is derived from pyruvate, links glycolysis to the TCA cycle, but is confined to the mitochondria. In the cytoplasm and nucleus, acetyl‐coenzyme A has to be derived by two alternative methods: first by ACLY, which utilizes citrate from the mitochondrial TCA cycle, and second by ACSS2, which ligates acetate to CoA. Acetyl‐coenzyme A can then be utilized as a cosubstrate by HAT, allowing transfer of the acetyl group to lysines on histone tails. 3‐PG, 3‐phosphoglyceric acid; SAH,* S*‐adenosylhomocysteine.

Metabolism and epigenetics are linked through the processes of methyl and acetyl transfer, utilizing *S*‐adenylmethionine (SAM) and acetyl‐coenzyme A as substrates respectively (Fig. 1). SAM is generated from the coupled folate and methionine cycles, collectively called one‐carbon metabolism. This is also essential for the synthesis of nucleotides, protein, lipids, and glutathione, which maintains the redox state of the cell. For this reason, it is often overactive in cancer 28, and is the reason why folate antagonists, such as methotrexate and pemetrexate, have proved to be successful in the clinic. Serine, glycine, methionine and folic acid are important sources for one‐carbon metabolism, and can be taken up by the cell. However, serine, glycine and methionine can be synthesized *de novo* in the cell, which helps to maintain one‐carbon metabolism when nutrients are scarce. Serine, which can be synthesized from 3‐phosphoglycerate, an intermediate in glycolysis, donates a carbon to the folate cycle while producing glycine, and converting tetrahydrofolate to methyltetrahydrofolate (mTHF). Glycine in turn, through the glycine cleavage system, can also provide one carbon, to produce mTHF. mTHF forms the link to the methionine cycle by providing the methyl group for betaine hydroxymethyltransferase to catalyse the reaction of homocysteine to methionine. Methionine can, in turn, be utilized in protein and lipid synthesis or adenylated to SAM, the major methyl donor in the cell. SAM is then utilized by DNA and HMTs to methylate amino acids on DNA and histone tails, respectively.

Acetylation of histones is dependent on the acetyl donor, acetyl‐coenzyme A. Acetyl‐coenzyme A is an important link between glycolysis and the TCA cycle in the mitochondria, and its formation is catalysed by pyruvate dehydrogenase. In the cytoplasm and nucleus, however, acetyl‐coenzyme A production is dependent on two different enzymes: ATP‐citrate lyase (ACLY) and acetyl‐coenzyme A synthetase short‐chain family member 2 (ACSS2) (Fig. 1) 29. ACLY produces acetyl‐coenzyme A from citrate, whereas ACSS2 ligates acetate to CoA. Acetyl‐coenzyme A then acts as the acetyl donor for lysine acetylation on histone tails by HAT. Whereas ACLY derives acetyl‐coenzyme A from the TCA cycle, ACSS2 is important as a scavenger of CoA from histone, protein and lipid deacetylation reactions. Interestingly, it may also utilize exogenous acetate, especially during hypoxia, as a source of acetyl‐coenzyme A 30, 31.

## Oncometabolites and their effect on the epigenome

**Figure 2:**
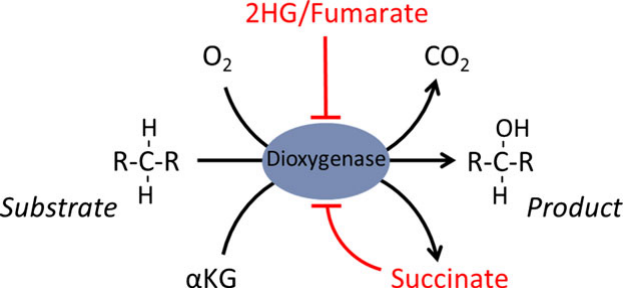
αKG‐dependent dioxygenases. This superfamily of enzymes uses oxygen and αKG as cosubstrates, resulting in the hydroxylation of the primary substrate and the decarboxylation of αKG, producing succinate and CO_2_. These enzymes can be inhibited by elevated levels of 2HG, succinate, or fumarate. This occurs by competition with the cosubstrate, αKG, and/or by product inhibition, as for succinate.

Several mutations in metabolic genes that lead to cancer formation have been identified over the last 15 years. These can promote epigenetic changes through a common mechanism, the accumulation of an ‘oncometabolite’, that acts as a competitive inhibitor of αKG‐dependent dioxygenases, an ever‐expanding superfamily of > 60 enzymes. Dioxygenases are involved in fatty acid metabolism, oxygen sensing, collagen biosynthesis, and modulation of the epigenome 32. Chemically, they all share a common requirement for oxygen and αKG as cosubstrates. Hydroxylation of the primary substrate occurs in conjunction with the oxidative decarboxylation of αKG to generate succinate and carbon dioxide (Fig. 2).

The first such effect was demonstrated for the loss of function of SDH in cancer. SDH loss was initially discovered in familial paraganglioma 33, but also occurs as a result of spontaneous somatic mutations 34. These tumours are of neuroendocrine origin and most commonly affect the carotid body, but can occur anywhere in the sympathetic and parasympathetic chain, as well as in the catecholamine‐secreting chromaffin cells in the adrenal gland; in this case, the tumour is called phaechromocytoma. SDH is a TCA cycle enzyme, and also complex II of the electron transport chain. It oxidizes succinate to fumarate, with the transfer of an electron to ubiquinone contributing to ATP production. The enzyme consists of four subunits, each of which can be mutated, causing a loss of function 33, 35, 36, 37.

**Figure 3:**
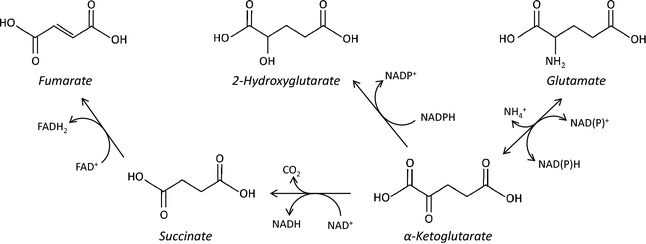
Metabolic structure of oncometabolites: Succinate, fumarate and 2HG are closely linked, both structurally and metabolically, to αKG. Succinate and fumarate differ from αKG only by the presence of a hydroxyl group on C2 and the loss of C1. In addition, succinate and fumarate differ only by the presence of an ethylenic bond, which may explain their similar tumorigenic effects. 2HG differs from αKG and glutamate only by the presence of a hydroxyl group instead of a ketone group or amine group, respectively. This explains how 2HG can competitively inhibit αKG by occupying the same enzymatic binding site.

FH catalyses the next reaction in the TCA cycle: the hydration of fumarate to malate. Similarly to SDH, loss of function of FH was also identified in a familial syndrome (hereditary leiomyomatosis and renal cell cancer) resulting in smooth muscle tumours called leiomyomas and aggressive renal cell carcinomas 38. Furthermore, FH loss have subsequently been identified, like SDH mutations, in paragangliomas and phaechromocytomas 39. In FH‐deficient and SDH‐deficient tumours, there is the respective accumulation of fumarate or succinate, which have a common effect by competitively inhibiting αKG‐dependent dioxygenases 40. It seems likely that succinate and fumarate, which are structurally similar, inhibit these enzymes through product inhibition, as the effects of both metabolites can be reversed by the addition of excess αKG *in vitro* and *in vivo* 41 (Fig. 3).

IDH is another TCA‐cycle related enzyme that has been implicated in tumorigenesis. On genomic screening, it was identified being commonly mutated in gliomas and in acute myeloid leukaemia (AML) 42, 43, 44. Interestingly, IDH mutations are associated with a better prognosis in glioma, but a poorer prognosis in AML 45. Subsequently, mutations have also been identified in other rare types of solid tumours, such as cholangiocarcinoma and chondrosarcoma 46, 47. IDH exists as three isoforms (IDH1, IDH2, and IDH3), with IDH1 being present in the cytoplasm and IDH2 and IDH3 in the mitochondria. IDH1 and IDH2 convert isocitrate to αKG by oxidative decarboxylation, with the production of NADPH from NADP^+^. IDH3 is structurally different from the other two isoforms, and utilizes NAD^+^ to produce αKG and NADH. It has not been found to be mutated in any cancer to date. The site of the mutation in IDH1 and IDH2 is at an equivalent arginine that is important to the active site for isocitrate binding 13. The mutation results in increased affinity αKG instead, and the mutated enzyme utilizes NADPH in a partially reversed reaction to produce a new metabolite, 2HG 13. 2HG is structurally very similar to αKG and glutamate, the only difference being the presence of a hydroxyl group instead of a ketone or amine group, respectively (Fig. 3). It is this similarity that results in the competitive inhibition of αKG‐dependent dioxygenases by 2HG, as it occupies the same binding site as αKG 48. Interestingly, the IDH1/IDH2 mutation is associated with a decrease in the level of αKG, potentially enhancing the inhibitory effect of 2HG on dioxygenases 49, 50.

A subgroup of αKG‐dependent dioxygenases implicated in tumorigenesis are the prolyl hydroxylases (PHDs), which play an important role in the degradation of hypoxia‐inducible factor (HIF) in normoxic conditions. HIF activates a range of genes in response to low oxygen, to increase glycolysis and angiogenesis. In the presence of oxygen, PHDs hydroxylate prolyl groups on HIF, allowing it to bind to the von Hippel–Lindau protein, which tags HIF for ubiquitylation and degradation in the proteasome 51. FH and SDH mutations cause a ‘pseudohypoxic’ phenotype through the inhibition of PHDs, which stabilizes HIF. This promotes a hypoxic response even in the presence of oxygen, causing increased glycolysis and angiogenesis 41, 52. The effect of 2HG on PHDs is less clear, with conflicting evidence of both inhibitory and activating effects 48, 53, 54, 55. This indicates variations between oncometabolites in their sensitivity to different members of the αKG‐dependent dioxygenase family of enzymes.

IDH, FH and SDH mutations are associated with changes in DNA and histone methylation. A subset of gliomas has been identified that has a distinct pattern of CpG island hypermethylation 17, which has been replicated in mutant IDH1‐overexpressing immortalized astrocytes, and a single‐copy IDH1 mutant knock‐in colorectal cell line 56, 57. IDH mutations in cholangiocarcinoma also replicate this hypermethylation pattern 58. This is also likely to extend to SDH mutations, with similar patterns of DNA methylation being observed in mouse‐derived SDH‐deficient chromaffin cells 59. The underlying mechanism for these changes seems to be inhibition of TET, an αKG‐dependent dioxygenase, which exists in three isoforms (TET1–TET3). They hydroxylate 5‐methylcytosine to 5‐hydroxymethylcytosine (5‐hmC), allowing subsequent DNA demethylation. In human glioma tissue samples, 5‐hmC levels are markedly reduced in IDH mutant as compared with IDH wild‐type tumours 48. In cell culture, this reduction in 5‐hmC levels has been replicated by overexpressing mutant IDH1/IDH2 in numerous cell lines, including glioma cells, immortalized astrocytes, and myeloblasts 56, 60. The same effect has also been observed in FH and SDH mutant cell models 40. Interestingly, in human AML samples, mutations in TET2 were found to be mutually exclusive to mutations in IDH, and to produce similar DNA methylation patterns to those in IDH mutant AML 60. Further genomic analysis of IDH1/IDH2 mutant AML revealed increased methylation of promoter sites of genes associated with myeloid differentiation, producing a more stem‐like phenotype 60. Overexpression of IDH1, or exposure to exogenous cell‐permeable 2HG, was able to promote cytokine independence and block differentiation in a leukaemic cell line. This could be replicated by knockdown of TET2, providing a potential link between 2HG, TET inhibition, and tumorigenesis 61.

The jumunji‐C HDMs are a subgroup of HDMs that are members of the α‐KG‐dependent dioxygenase family 32. They initiate the first step in the removal of methyl groups by hydroxylation, causing an increase or decrease in gene transcription, depending on the histone methylation site. Jumunji‐C HDMs are very sensitive to high levels of fumarate, succinate, and 2HG 62. In the case of the IDH1/IDH2 mutation, increases in histone methylation have been observed in human glioma samples for H3K9 and H3K27, both of which are gene repressive marks 48, 63. Interestingly, the IDH1 mutation was strongly correlated with the level of trimethylated H3K9 (H3K9me3) in oligodendrogliomas but not in astrocytomas, which are two different subtypes of glioma 64. This implies that there is a differential effect of 2HG on different tumours even in the same tissue type. Murine 3T3‐L1 cells, which can be differentiated into adipocytes, were used to prove a link between the repressive histone methylation marks H3K9me3 and trimethylated H3K27 (H3K27me3), and cellular differentiation. Overexpression of mutant IDH1 was associated with impaired adipogenesis resulting from increases in both H3K9me3 and H3K27me3 at promoter sites for transcription factors responsible for adipocyte differentiation. Similar changes in histone methylation markers, and impaired differentiation, were also seen in IDH1‐overexpressing primary murine neurospheres 63. Increases in H3K27me3 and H3K9me3 levels have also been observed in SDH and FH mutant tumours and cell models 40, 59. Interestingly, increased levels of H3K9me3 occur prior to increases in DNA methylation when mutant IDH1 is introduced into immortalized astrocytes 63. These histone methylation changes may therefore account for some of the changes in DNA methylation patterns 65.

## Future therapies

There is now increasing evidence that mutations in metabolic enzymes are, in part, responsible for the epigenetic changes in some cancers. At least in AML, there is good evidence to suggest that the IDH1 mutation alone may be sufficient to induce leuko‐neogenesis by inhibiting genes responsible for cell differentiation through DNA hypermethylation 61. For other tumours, this is less clear, but, at least in glioma, the IDH mutation seems to be an early event that is maintained throughout tumour progression 42. It is also becoming evident that the presence of oncometabolites in tumours is not confined to malignancies with TCA gene mutations. Elevated levels of 2HG, driven by myc activation, have been identified in breast cancer, resulting in DNA hypermethylation 66. Interestingly, 3‐phosphoglycerate dehydrogenase, which is the enzyme responsible for the first step in serine biosynthesis from the glycolytic intermediate 3‐phosphoglycerate, has recently been shown to convert αKG to 2HG by utilizing NADH 67. This provides a possible link between increased myc‐driven glycolysis and 2HG production in some breast cancers. It seems likely that other tumours may also be affected, and new oncometabolites may be identified in the future. In fact, an *in silico* systems approach using 1700 genomes has already been used to identify potential new oncometabolites in a range of tumours 68.

The discovery of metabolic enzymes that can alter the epigenome has opened up a new, exciting area for drug development. In only 5 years, IDH1 and IDH2 small‐molecule inhibitors have been developed that are now entering clinical trials. AGI‐5198, an IDH1 inhibitor, was tested in a heterozygous IDH1 mutant glioma cell line (TS603). It was able to reverse H3K9 trimethylation, promote cellular differentiation, and delay growth, although, interestingly, it had no effect on DNA methylation 69. Similarly, AGI‐6780, a specific inhibitor of mutant IDH2, induced leukaemic cell differentiation in primary human samples *ex vivo* 70. Further investigation using a mutant IDH2‐overexpressing leukaemic cell line showed reversal of both DNA and histone hypermethylation, inducing cell differentiation. Interestingly, histone methylation is rapidly reversed within days, whereas DNA methylation progressively changes over a period of weeks 71. This may account for the lack of change in DNA hypermethylation seen with AGI‐5198.

It will be interesting to determine whether these new drugs are equally efficacious in different types of IDH‐mutated tumour. In glioma, the presence of an IDH1 mutation is actually associated with better prognosis than that of wild‐type tumours. These tumours grow more slowly *in vitro* and *in vivo* 72. The concern in glioma is that inhibiting 2HG production may potentiate tumour growth. Reduced expression of branched‐chain amino acid transferase 1 (BCAT1) in IDH1‐mutated gliomas has been shown, in part, to be caused by hypermethylation of the BCAT1 promoter region. When BCAT1 is overexpressed in IDH1‐mutant immortalized human astrocytes, some of the loss in cell proliferation is recovered 73. This raises the concern that inhibition of the mutant IDH enzyme in glioma may increase cell proliferation, and we may need to focus on specific downstream pathways affected by 2HG. This is in direct contrast to AML, in which IDH mutations are associated with a worse prognosis and more aggressive disease, and in which mutant IDH inhibitors may prove more beneficial.

## Author contributions

Stefan Nowicki and Eyal Gottlieb reviewed the literature and wrote the manuscript.
